# Simultaneous occurrence of cutaneous melanocytic neoplasia in Duroc and German Saddleback pigs from three smallholder farms

**DOI:** 10.1186/s12917-025-04863-0

**Published:** 2025-06-27

**Authors:** Thies J. Nicolaisen, Melanie Stoff, Manon Mikic, Anna Lena Maschmeier, Johannes Buchallik-Schregel, Alexandra von Altrock, Maybrit Wirtz, Saskia Neubert, Andreas Beineke, Doris Höltig, Isabel Hennig-Pauka, Martin Ganter

**Affiliations:** 1 Clinic for Swine and Small Ruminants, Forensic Medicine and Ambulatory Clinic, University of Veterinary Medicine Hannover, Foundation, Hannover, Germany; 2 Department of Pathology, University of Veterinary Medicine Hannover, Foundation, Hannover, Germany; 3 Department of Small Animal Medicine and Surgery, University of Veterinary Medicine Hannover, Foundation, Hannover, Germany; 4 Field Station for Epidemiology (Bakum), University of Veterinary Medicine Hannover, Foundation, Hannover, Germany; 5 Present Address: Institute for Animal Hygiene, Animal Welfare and Farm Animal Behaviour, University of Veterinary Medicine Hannover, Foundation, Hannover, Germany

**Keywords:** Duroc, German Saddleback, Husum Red Pied, Congenital, Neoplasia, Melanoma, Melanocytoma, Broncho-alveolar lavage, Small-scale farms

## Abstract

**Background:**

Congenital melanocytic neoplasia is a rare disease that is described for the pig breeds Duroc, Iberico and Nero Siciliano used in agriculture. The hereditary nature of this neoplastic condition has been substantiated. The clinical course is unpredictable, spanning from complete spontaneous regression of primary tumors and survival of afflicted animals to a fatal progression marked by metastases to internal organs. In contrast to other species, no clinical or histological prognostic criteria for assessing the clinical course of disease exist in pigs.

**Case presentation:**

Four cases of cutaneous melanocytic neoplasia are described that occurred concurrently in three small-scale farms in northwestern Germany in 2023. These cases underwent thorough diagnostic evaluation employing a spectrum of modalities including clinical examination, hematological analysis, pathohistological examination, X-ray imaging, broncho-alveolar lavage, computed tomography, and autopsy. The first and the second case deal with two Duroc crossbred littermates with divergent courses of disease: While the black colored piglet had to be euthanized at eight weeks of life due to widespread metastases affecting multiple organs, the neoplastic disease remained localized to the primary tumor in the red-brown colored pig. Broncho-alveolar lavage was utilized in the black colored piglet to identify metastatic lesions of the melanoma within the lung tissue. The third case represents the initial observation of an affected Duroc gilt manifesting generalized leukoderma and leukotrichia during the regression phase of the cutaneous melanocytic tumor. The first observation of a congenital melanoma in a German Saddleback (variety: Husum Red Pied) is described in the fourth case. Notably, the primary tumor exhibited regression over the course of the disease.

**Conclusion:**

A higher incidence of this neoplastic disease can be assumed for small-scale farms in northwest Germany compared to professional pig husbandry due to potential inbreeding. No epidemiological or family link could be found between the two cases of affected Duroc pigs. Notably, the endangered Husum Red Pied breed maintains a limited genetic diversity, emphasizing the imperative of integrating considerations of this disease into breeding programs to mitigate the heightened risk of hereditary neoplastic afflictions in subsequent generations.

**Supplementary Information:**

The online version contains supplementary material available at 10.1186/s12917-025-04863-0.

## Background

Cutaneous melanocytic neoplasms are tumors of the pigment producing cells (melanocytes) and can occur as benign melanocytoma or malignant melanoma. Congenital occurrence is a special type of a cutaneous melanocytic tumor and described in several farm animal species, e.g. pigs [1], cattle [2], horses [3] and goats [4]. In pigs, these tumors are described in the agricultural pig breeds Duroc [5], Iberico [6] and Nero Siciliano [7]. Several case reports of congenital melanoma in Duroc breed exist throughout the late twentieth century [8–10] after which they decreased. An inheritance has been confirmed for this disease [1, 11]. Despite the hereditary basis, congenital melanoma is a sporadically occurring disease in farmed pigs [12] and case reports of the recent century are rare. The Sinclair Miniature Pig [13, 14], Munich Miniature Pig “Troll” [15] and Melanoblastoma Bearing Libechov Miniature Pig (MeLiM) [16, 17] were bred for this genetic trait and serve as model animals for melanoma research due to a similar pathology to human melanoma [18].

## Case presentation

### Farm description and Anamnesis

Three small-scale farms that were located in northwest Germany were affected by cutaneous melanocytic lesions in autumn/winter 2023/2024. Two of them kept Duroc pigs, either purebred Duroc (Farm DuP) or crossbred Duroc (Farm DuC). The third farm (Farm HRP) kept the indigenous breed German Saddleback (variety Husum Red Pied) and bred within a herd book organization. Detailed information about the affected farms can be found in Table 1. While the farms DuC and HRP reported about affected piglets in recent litters (Farm DuC: *n* = 2; Farm HRP: *n* = 1) that exhibited congenital cutaneous melanocytic lesions, the farm DuP reported about one affected pig that was purchased from a third farm after weaning. All pigs were referred to the Clinic for Swine and Small Ruminants of the University of Veterinary Medicine Hannover for further investigations between November 2023 and January 2024.

**Table 1:** Farm descriptions of the three affected small-scale farms

Farm	Farm DuC (Duroc crossbred)	Farm DuP (Duroc purebred)	Farm HRP (Husum Red Pied)
Breed	Duroc crossbred (mother sow of affected litter: 15/16 Duroc, 1/16 German Saddleback (variety: Angeln Saddleback))	Duroc purebred	German Saddleback (variety: Husum Red Pied)
Breeding stock	1 breeding sow, 1 gilt, 1 boar	2 breeding sows, 4 gilts, 1 boar	1 breeding sow
Herd book breeding	No	No	Yes
Replacement of breeding stock	Breeding of own replacement gilts; adult boar purchased from a third small-scale-farm (December 2022)	Purchase of replacement gilts from a third small-scale farm	Breeding of own replacement gilts
Natural mating / Artificial insemination	Natural mating	Artificial insemination (semen purchased from a professional breeding company)	Natural mating (boar prescribed by the herd book organization)
Affected pigs with cutaneous melanocytic lesion	Two piglets affected (one brown and one black piglet) in a litter with 13 total born piglets; born October 2023	One affected gilt (purchased after weaning with three unaffected female littermates); born August 2023	One piglet affected in a litter out of 11 total born piglets; born in October 2023
History of cutaneous melanocytic lesions on the farm	No	Two piglets were born on the farm before (within 18 months before the presented case)	No

## Clinical examination

**Fig. 1:**
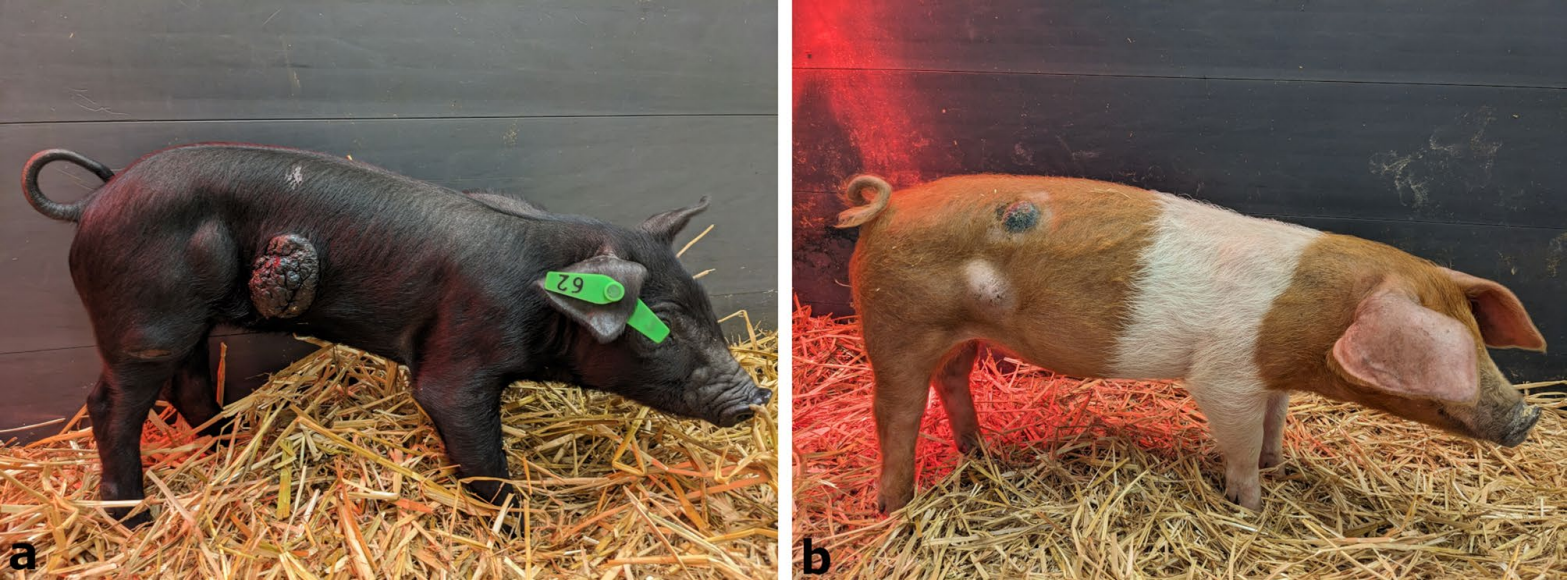
**a**-**b**: Black DuC-pig (6th week of age) and HRP-pig (10th week of age) both with congenital melanoma on their right flanks and enlarged right subiliac lymph nodes

**Fig. 2:**
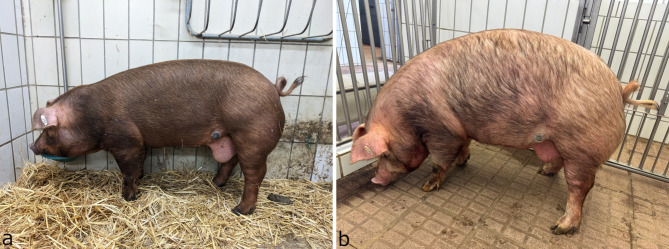
**a**-**b**: Clinical signs of leukoderma and leukotrichia in the DuP-pig. **a**: Onset in the 23rd week of age; **b**: 35th week of age

Clinical signs of the initial clinical examination of the four affected pigs can be found in Table 2. The complete findings of the clinical examinations can be found in Table A1.1.1 (red-brown DuC-pig, DuP-pig and HRP-pig) and in Table A1.1.2 (black DuC-pig) of the Additional file 1. All four patients showed at least one cutaneous melanocytic lesion. The character of these lesions differed among the pigs from exophytic (*n* = 1; black piglet from Farm DuC [Figure 1a]) and nodular (*n* = 2; brown piglet from Farm DuC, pig from Farm HRP [Figure 1b]) to macule-like (*n* = 1, pig from Farm DuP, [Figure 2a-b]). In two pigs (black piglet from Farm DuC and pig from Farm HRP) two or more cutaneous melanocytic lesions were found. Enlarged regional lymph nodes were suspected in three pigs during the clinical examination (*Lymphonodus* (*Ln.*) *subiliacus*: black pig from Farm DuC, pig from Farm HRP; *Ln. inguinalis superficialis*: black pig from Farm DuC, pig from Farm DuP). Growth retardation was suspected in two pigs (black piglet from Farm DuC, pig from Farm DuP).

**Table 2:** Clinical signs found in the initial clinical examination of the four pigs

Pig	DuC: red-brown piglet	DuC: black piglet	DuP-pig	HRP-pig
Gender and Age	Female, six weeks of life	Female, six weeks of life	Female, 23rd weeks of life	Female, seven weeks of life
Body weight and Body Condition Score (BCS)	9.8 kg; BCS: 3	6.7 kg; BCS: 2	73 kg; BCS: 3	16.2 kg; BCS: 3
Cutaneous melanocytic lesions	Forehead: nodular growth of the skin: diameter 4 cm, height 2 cm, black, solid, dry, hairless, central bright area	Right flank: exophytic growth of the skin: diameter 4–6 cm, height 1 cm, black, hairless, covered with scab Right hind limb: nodular growth proximal to the lateral dew claw: diameter 1 cm, black, solid, dry, hairless	Left, caudoventral flank: macule-like melanocytic lesion: diameter 3 cm, black, macule-like, dry, hairless, skin excessed and wrinkled	Right flank of the body at the level of the transverse processes of the lumbar spine: nodular growth, diameter 3 cm, height 1 cm, black, solid, dry, hairless, central bright area; macules and nevi on multiple parts of the body
Lymph nodes	-	Right inguinal lymph node: moderate enlargement (diameter 1 cm) without signs of inflammation Right subiliac lymph node: severe enlargement (4 × 3 × 1,5 cm) without further signs of inflammation	Left inguinal lymph node: severe enlargement (10 × 5 × 5 cm) without signs of inflammation	Right subiliac lymph node: severe enlargement (5 × 2 × 2 cm) without signs of inflammation
Respiratory tract	Respiratory rate: 24 breaths/minute; breathing type: costo-abdominal, coughing was triggerable by coupage of the body	Respiratory rate: 24 breaths/minute; breathing type: costo-abdominal	Respiratory rate: 32 breaths/minute; breathing type: costo-abdominal	Respiratory rate: 30 breaths/minute; breathing type: costo-abdominal

Abbrevations: DuC: Duroc crossbred; DuP: Duroc purebred; HRP: Husum Red Pied

## Course of disease

### Farm DuC, red-brown pig

A cutaneous melanocytic neoplasia of the skin was diagnosed in the histopathological examination. Radiographic examination of the thorax, computed tomographic examination of the head, thorax and abdomen and a broncho-alveolar lavage (BAL) were performed and no evidence for metastases was found. The pig developed normally during hospitalization (observation period: 6th to 40th week of life). General clinical examinations were conducted on a regular basis in which no indication of metastatic spread was found, e.g. enlargement of lymph nodes or retarded growth. An increase in the size of the tumor occurred over time. The color of the skin tumor changed over time and parts of the tumor became amelanotic (Additional Figure A1a-c). The other characteristics of the skin tumor did not change. The first signs of regression in form of local (around the primary tumor) and general leukotrichia (hams) became visible in May 2024 (31st week of life). By July 2024 (40th week of life) the pig was in a general good condition. Neither signs of metastatic spread nor regression of the primary tumor were seen. The dimensions of the primary tumor were approximately 9 cm x 9 cm and its height approximately 3.5 cm, leukoderma and leukotrichia progressed and a discoloration of the former brown-colored iris to a blue-grey color was observed.

### Farm DuC, black pig

**Fig. 3:**
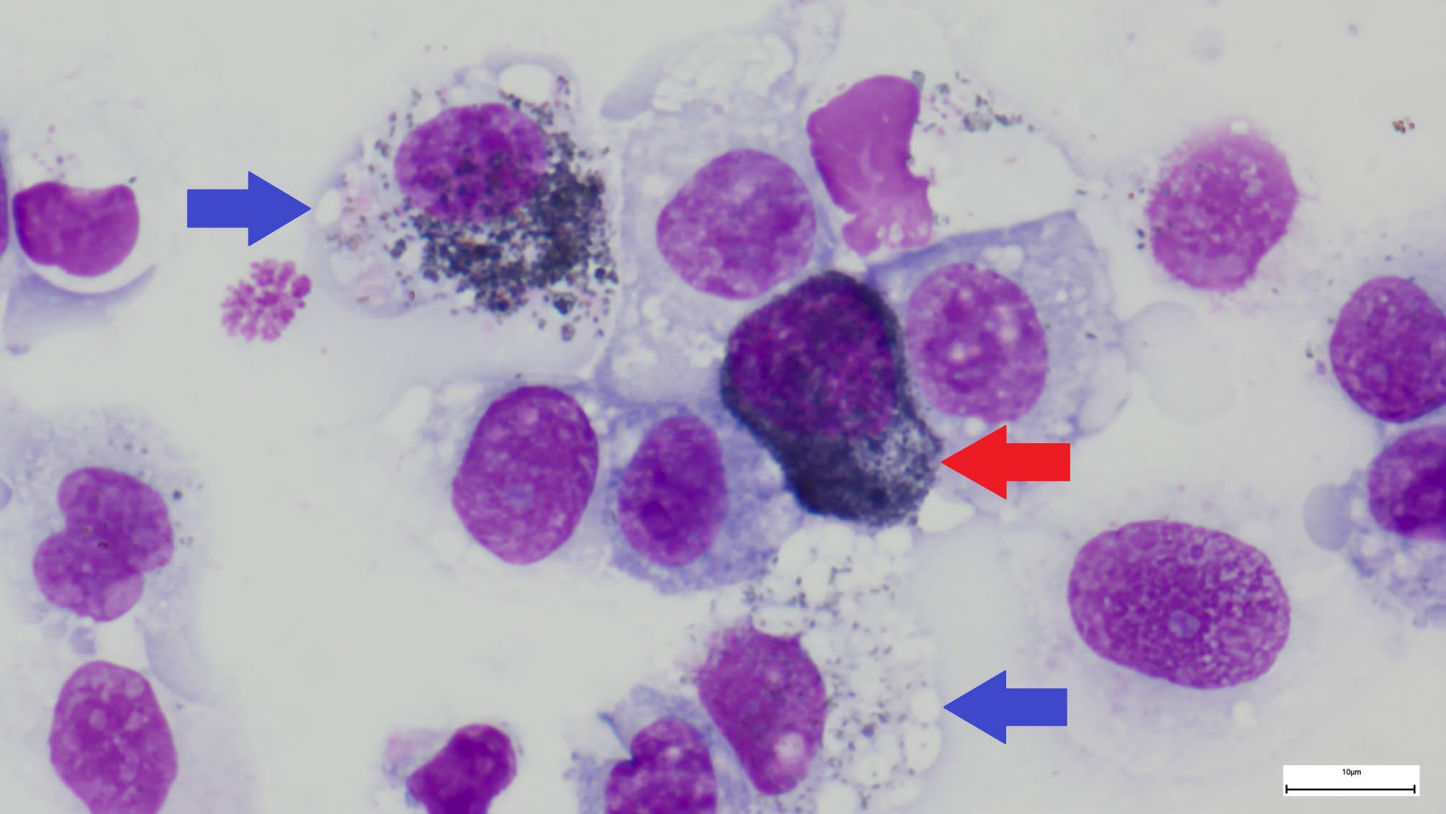
The cytology of the BALF of the black DuC-pig contained numerous cells with variable amounts of brown-black granules (melanin). On the one hand, pigmented cells showed a polygonal morphology with additional cytoplasmic vacuolation and an eccentric located nucleus with slightly granular chromatin (presumably melanophages, blue arrow). In addition, a second population of pigmented cells is present with polygonal shape, high content of melanin granula that partly obscures nuclear details and a large, eccentric located nucleus (likely melanocytes, red arrow). Pappenheim stain, scale bar = 10 μm

Histopathological samples (primary tumor and lymph node) exhibited evidence of melanocytic neoplasia. A first radiographic examination of the thorax was performed shortly after hospitalization and revealed multiple round, soft tissue opaque mass lesions within the lung parenchyma. A BAL was performed under general anesthesia and the cytological examination supported the suspicion of lung metastases (Fig. 3).

**Fig. 4:**
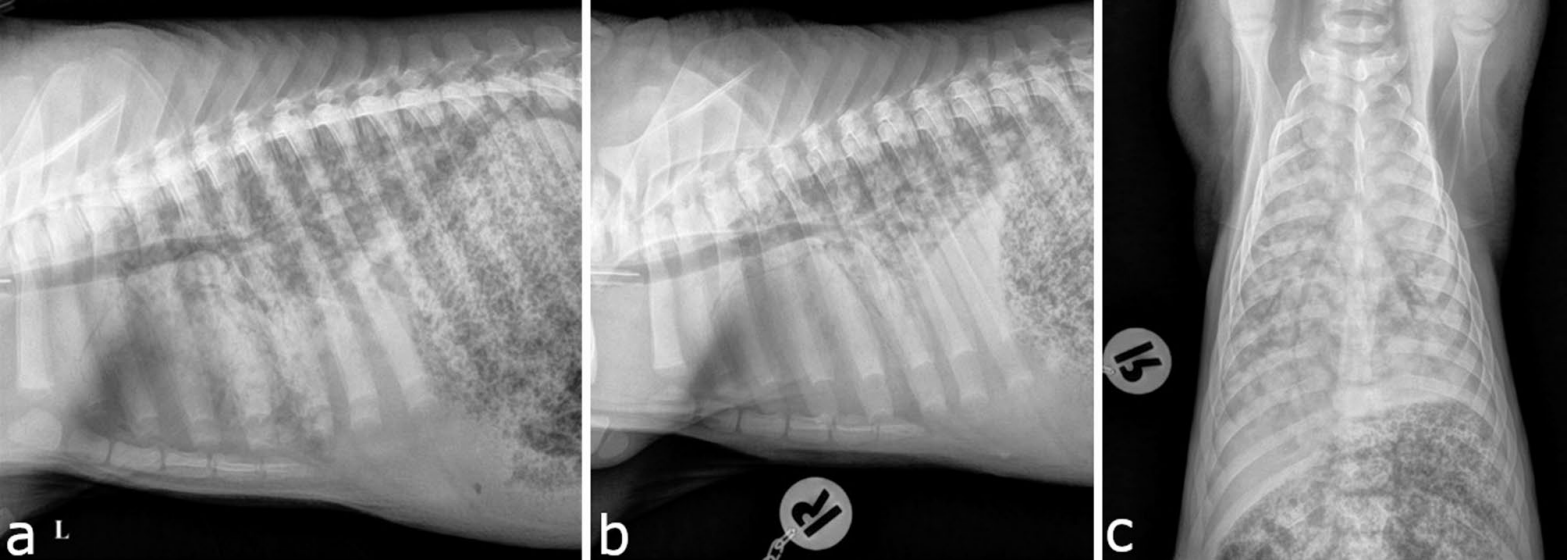
**a**-**c**: Left lateral, right lateral and ventrodorsal projection of the thorax of the black DuC pig. Note the generalized increased radiopacity and presence of air bronchograms. There is almost complete border effacement of the cardiac silhouette due to soft tissue opacity of the lung parenchyma

**Fig. 5:**
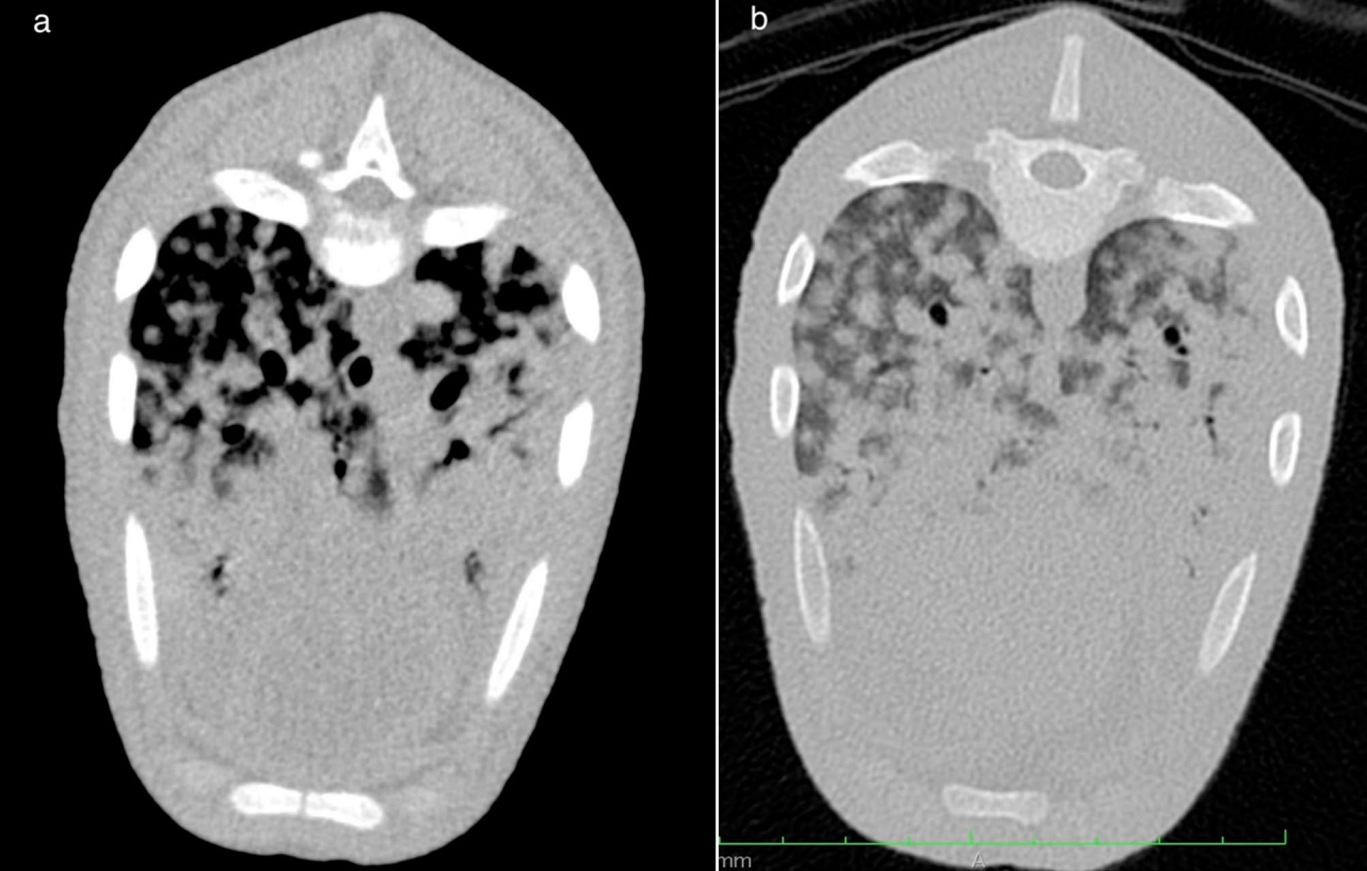
a-b: Black DuC-pig: Transverse computed tomography images at the level of the caudal cardiac silhouette, using soft tissue reconstruction (**a**) and lung reconstruction (**b**) algorithms. The left side of the image is the right side of the patient. Note the nodular lesions of soft tissue attenuation and the generalized increased attenuation of the lung parenchyma

The pig’s state of health was checked on a daily basis and seventeen days after hospitalization (9th week of life) the general condition of the piglet began to worsen. A comprehensive clinical assessment was performed, revealing deviations from normal behavior, including apathy characterized by diminished interest in the environment, social withdrawal from conspecifics, and an increased tendency to remain in a lying position. The respiratory rate was 24 breaths per minute, and a mild inspiratory breathing sound could be heard on the upper lung border on both body sides. All findings of the three clinical examinations that were conducted during hospitalization and the daily measured rectal temperature can be found in Table A1.1.2 and Table A1.2.1 of the Additional file 1. A second radiographic examination (Fig. 4a-c) and a computed tomography of the head, thorax (Fig. 5a-b) and abdomen were conducted on the same day and revealed multiple metastases in the internal organs. The decision to euthanize the pig was made due to ethical reasons and the unfavorable prognosis. Afterwards, the piglet was sent to the Department of Pathology for necropsy and the diagnosis of a melanoma with multiple metastases in internal organs was confirmed (see subsection 4.2.2).

### Farm DuP-pig

The histopathological examination revealed melanocytic neoplasia of the skin and metastasis in the inguinal lymph node. No evidence for lung metastases were found in the radiographs of the lungs nor the broncho-alveolar lavage fluid (BALF). First clinical signs of leukoderma and leukotrichia on the face, the back, the flanks and the hind leg and a blue-grey color of the iris became evident three weeks after hospitalization (26th week of life). The leukoderma and leukotrichia progressed to an almost complete depigmentation of iris, skin and bristles (Fig. 2a-b). In July 2024, (50th week of life) the pig was in a good general condition and there were no signs of further metastases. However, neither the diameter of the primary tumor, nor the size of the affected inguinal lymph node decreased in size over time.

### Farm HRP-pig

Histopathological examination of the cutaneous melanocytic lesions and the enlarged subiliac lymph node revealed evidence for a melanoma of the skin with associated metastasis in the subiliac lymph node. Radiographic examination, computed tomography and BAL were performed, but no evidence for further metastatic spread in the body of the pig was found. During the course of disease, the melanoma of the skin flattened (Additional Figure A2a-b). It remained black and dry, the surface became scaly and encrusted. The size remained at approximately 7 cm in diameter. There was no change in the size of the subiliac lymph node over time. The pig developed signs of local leukotrichia around the primary tumor (20th week of life) followed by generalized onset of leukoderma and leukotrichia. As of July 2024, the pig was in good general condition and no clinical signs for a progression of the disease were evident.

## Diagnostics

### Hematological examination

Hematological examinations were carried out in all four pigs after hospitalization. The complete results of the hematological examinations for all four pigs can be found in Table A1.3.1 of Additional file 1. Breed specific reference ranges used for interpretation were evaluated by Nerbas [19]. The following pathological findings were revealed by hematological examination: black DuC-pig: Packed cell volume (0.33 l/l) in the lower reference range and a moderate neutrophilia with a severely increased proportion of segmented neutrophils (16.01 G/l); A mild neutrophilia with a slight increased proportion of segmented neutrophils was evaluated for DuP-pig (8.42 G/l) and HRP-pig (8.7 G/l). No abnormal results were found for the red-brown DuC-pig.

### Punch biopsies, histopathology and necropsy

Punch biopsies were taken under general anesthesia (ketaminhydrochlorid 20 mg/kg body weight intramuscularly [Ketamin 100 mg/ml, cp. pharma, Burgdorf, Germany], azaperone 2 mg/kg body weight intramuscularly [Sedanol 40 mg/ml, WDT, Garbsen, Germany]) from the cutaneous melanocytic lesions and the enlarged regional lymph nodes of all pigs (anesthesia protocols for the different examinations: see Additional file 1, A.1.6). Meloxicam (0.4 mg/kg body weight intramuscularly; Melosolute 20 mg/ml, cp. pharma, Burgdorf, Germany) was used for pain management shortly before and after the biopsy procedure. A black coloration of all affected lymph nodes (black DuC-pig, DuP-pig, HRP-pig) became visible during the biopsy procedure. The biopsies were sent for histopathological examination to the Department of Pathology, University of Veterinary Medicine, Hannover, Foundation. The preparation of histopathological samples and immunohistochemistry are described in the Additional file 1, A 1.7.

#### Red-brown DuC-pig

Within the superficial dermis and extending into the subcutis parts of a cell-rich, highly pigmented neoplastic mass with reduced degree of pigmentation towards the subcutis was present. Neoplastic cells were arranged in sheets supported by minimal fibrovascular stroma. The cells were medium-sized to large, with either round to polygonal or spindeloid shape, scant to moderate amount of eosinophilic, granular cytoplasm that contained variable amounts of brown-black, granular pigment that discolored after bleaching (melanin) and indistinct cell borders. They contained a central located, round to oval, medium-sized nucleus with finely stippled chromatin and up to two prominent basophilic nucleoli. There was moderate anisocytosis and anisokaryosis and the mitotic rate was 0 per 2.37 mm^2^. Immunohistochemically, moderate numbers of tumor cells showed positive immunolabelling for MelanA and PNL2 as markers for melanocytes. Additionally, highly pigmented Iba1^+^-melanomacrophages were present.

#### Black DuC-pig

Biopsies of the skin and subcutaneous area of the regional lymph node contained parts of a cell-rich, variable pigmented neoplastic mass. Neoplastic cells were arranged in sheets supported by minimal fibrovascular stroma. The cells were medium-sized to large, with either round to polyogonal or spindeloid shape, scant to moderate amount of eosinophilic, granular cytoplasm that contained variable amounts of brown-black, granular pigment that discolored after bleaching (melanin) and indistinct cell borders. They contained a centrally located, round to oval, medium-sized nucleus with finely stippled chromatin and up to two prominent basophilic nucleoli. There was moderate anisocytosis and anisokaryosis and the mitotic rate was 1 per 2.37 mm^2^.

**Fig. 6:**
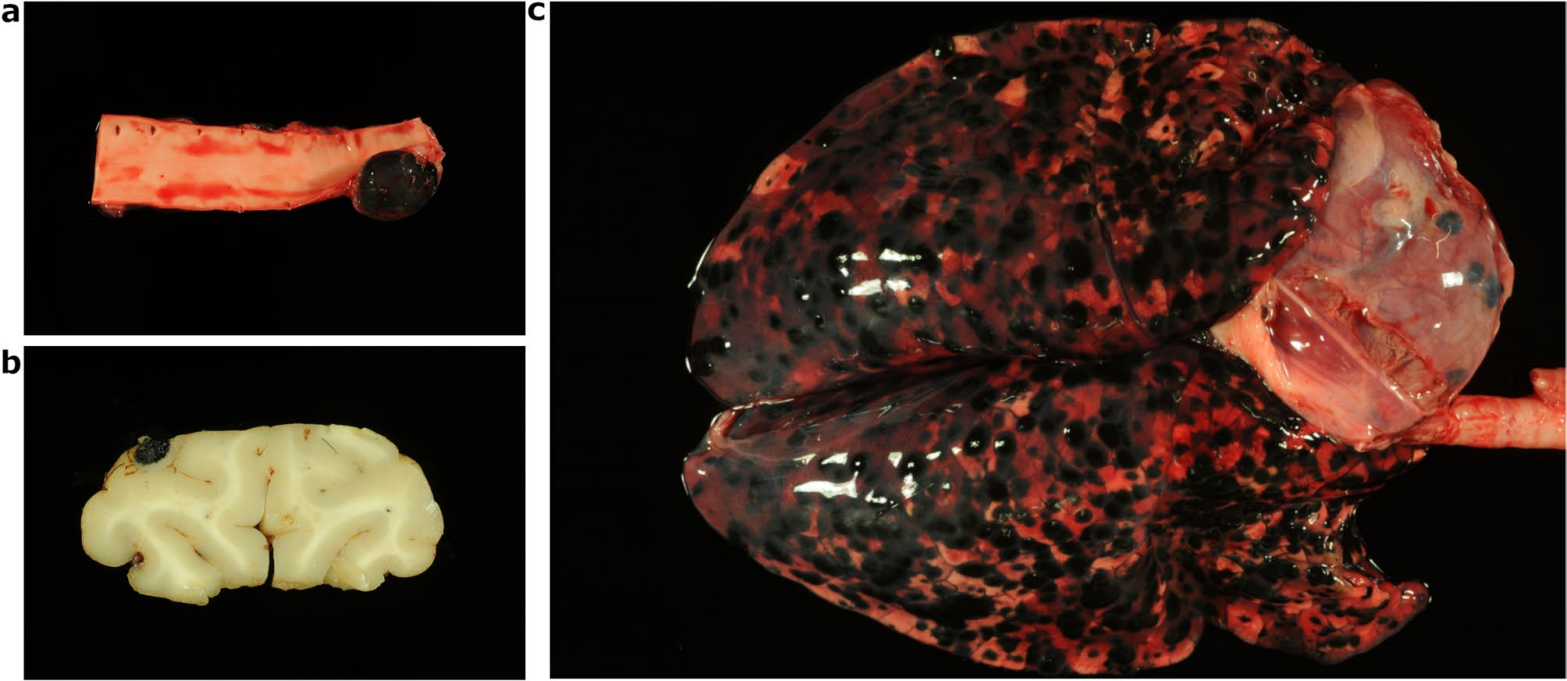
**a**-**c**: Metastases of the melanoma of the black DuC-pig in one *Ln. thoracicus aorticus* (**a**), the cerebral cortex (b) and lung and pericardial sac (**c**)

At necropsy, the examined piglet showed an exophytic, well demarcated, round to ovoid, black-colored, hairless and superficially irregular fissured, 4 × 4 × 1 cm measuring melanoma in the skin on the right flank. Numerous metastases (Fig. 6a-c) ranging from 1 mm in diameter up to 3.5 × 6 × 3.5 cm were present in the subcutis, lymph nodes (*Lymphonodus* [*Ln.*] *tracheobronchialis dexter*, *Lymphonodi* [*Lnn.*] *subiliaci*,* Lnn. renales*, *Ln. cervicalis superficialis sinister*, *Ln. inguinalis superficialis*, *Lnn. thoracici aortici*), skeletal muscles, tongue, esophagus, stomach, pancreas, spleen, subtonsillar pharynx, pericardial sac, lung (disseminated), thymus, liver, intestine, kidneys, bone (*Condylus occipitalis*, multiple vertebral bodies), inner ear and central nervous system (cerebral cortex, cerebellum, brain stem, cervical spinal cord).

**Fig. 7:**
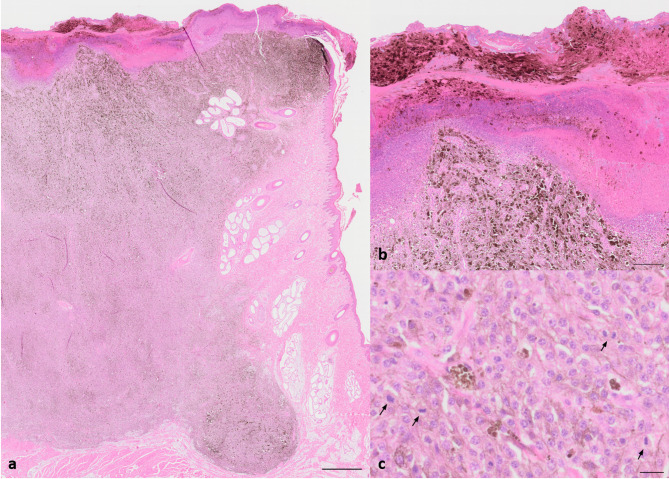
**a**-**c**: Black DuC-pig, cutaneous melanoma right flank. **a**: Exophytic, ulcerated, non-encapsulated cutaneous melanoma with variable degree of pigmentation and extension into subcutis. Hematoxylin and eosin stain, scale bar = 1 mm. **b**: Extended superficial ulceration with loss of adnexal structures, formation of serocellular crusts, intralesional bacterial colonies and neutrophils. Hematoxylin and eosin stain, scale bar = 200 μm. **c**: Tumor cells are supported by delicate strands of fibrovascular stroma. They are arranged in sheets, medium-sized to large, round to polygonal with moderate amount of cytoplasm that contains variable amounts of brown-black, granular pigment. A mitotic rate of 12 mitoses (arrows) per 2.37 mm^2^ was present. Hematoxylin and eosin stain, scale bar = 20 μm

**Fig. 8:**
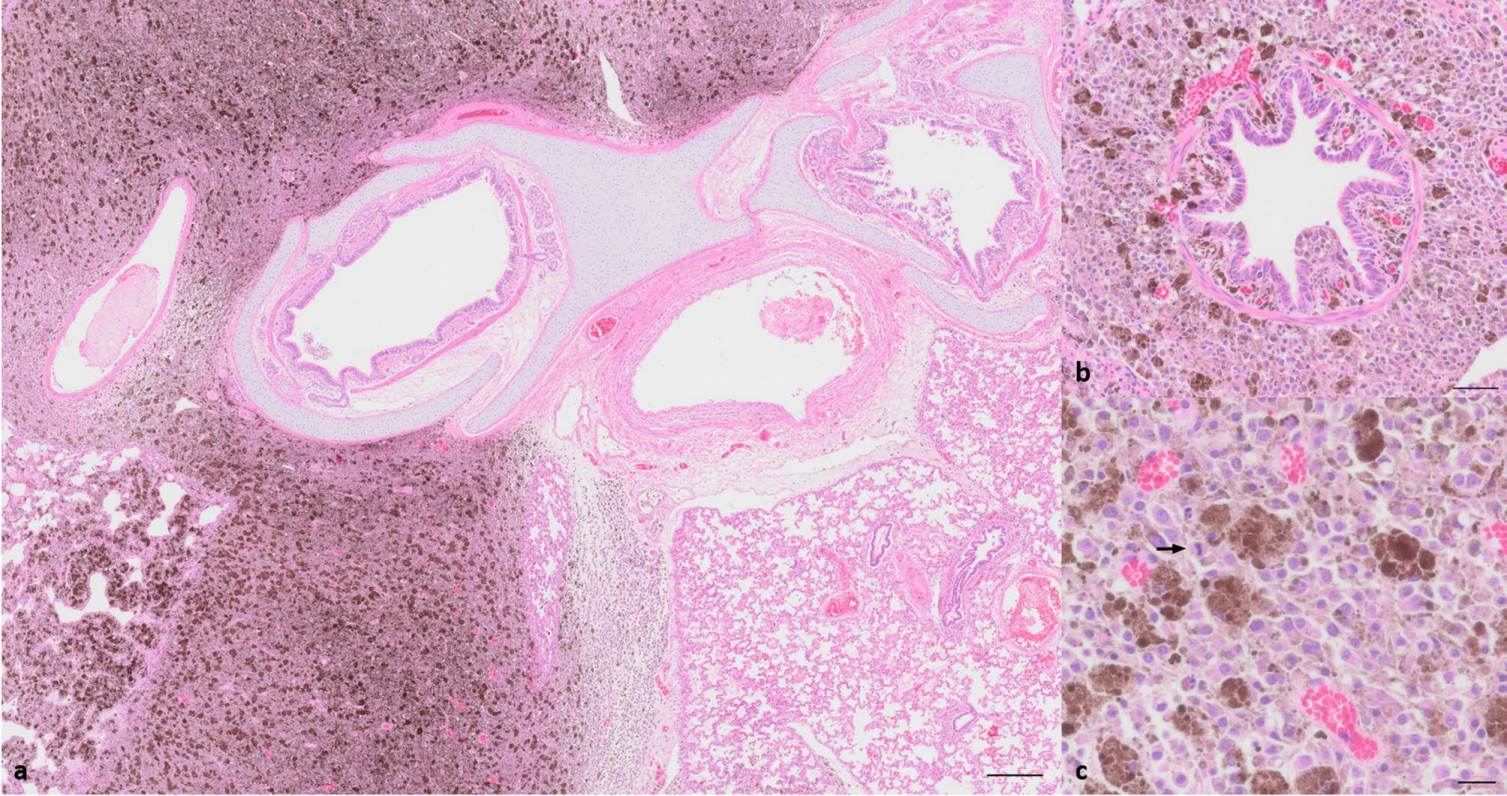
**a**-**c**: Black DuC-pig, metastatic melanoma lung. **a**: Extensive infiltration of variable pigmented tumor cells and melanomacrophages within the lung parenchyma, partly filling alveolar spaces and expanding perialveolar and interlobular interstitium. Hematoxylin and eosin stain, bar = 250 μm. **b**: Bronchioli and peribronchiolar space with infiltration of variable pigmented tumor cells and melanomacrophages. Hematoxylin and eosin stain, scale bar = 50 μm. **c**: Pulmonary tumor cell infiltrates are arranged in sheets, medium-sized to large, round to polygonal with moderate amount of cytoplasm that contains variable amounts of brown-black, granular pigment. Occasionally, mitotic figures (arrow) are present. Hematoxylin and eosin stain, scale bar = 20 μm.

Histologically, all tumors (Fig. 7a-c: cutaneous melanoma; Fig. 8a-c: lung metastases; Additional Figure A3: cerebral metastases) presented as cell-rich, non-encapsulated, poorly demarcated, infiltrative masses with variable degree of pigmentation. Neoplastic cells were arranged in streams, bundles and sheets supported by minimal fibrovascular stroma. The cells were medium-sized to large, with either round to polygonal or spindeloid shape, scant to moderate amount of eosinophilic, granular cytoplasm that contained variable amounts of brown-black, granular pigment that discolored after bleaching (melanin) and indistinct cell borders. They contained a centrally located, round to oval, medium-sized to large nucleus with finely stippled chromatin and up to two prominent basophilic nucleoli. There was moderate anisocytosis, anisokaryosis and anisonucleolosis. Within the cutaneous melanoma (Fig. 7a-c) a mitotic rate of 12 mitoses per 2.37 mm^2^ was present and the tumor showed an extended superficial ulceration with formation of serocellular crusts, intralesional bacteria and infiltration of viable and degenerated neutrophils. Frequently, single mitotic figures were observed in the other tumor locations. Immunohistochemically, moderate numbers of tumor cells showed positive immunolabelling for MelanA and PNL2. All tumors contained Iba1^+^-melanomacrophages.

#### DuP-Pig

The biopsy of the skin contained parts of a moderately cellular, highly pigmented neoplastic mass within the dermis. Neoplastic cells were arranged in sheets and supported by scant amount of fibrovascular stroma. Cells were medium-sized to large, round to polygonal with moderate to large amount of brown-black, granular pigmented that discolored after bleaching (melanin) cytoplasm. They contained a centrally located, round to oval, medium-sized nucleus with finely stippled chromatin and one basophilic nucleolus. There was mild anisocytosis and anisokaryosis and a mitotic rate of 0 mitoses per 2.37 mm^2^.

The biopsy of the inguinal lymph node showed multifocal follicular aggregates of lymphocytes and an infiltration of a moderately cellular, highly pigmented neoplastic mass. Neoplastic cells were arranged in sheets supported by minimal fibrovascular stroma. The cells were medium-sized to large, with either round to polygonal or spindeloid shape, scant to moderate amount of eosinophilic, granular cytoplasm that contained variable amounts of brown-black, granular pigment that discolored after bleaching (melanin) and indistinct cell borders. They contained a centrally located, round to oval, medium-sized nucleus with finely stippled chromatin and up to two basophilic nucleoli. There was moderate anisocytosis and anisokaryosis and a mitotic rate of 0 mitoses per 2.37 mm^2^.

Immunohistochemically, moderate numbers of cutaneous and nodal tumor cells showed positive immunolabelling for MelanA and PNL2. Additionally, highly pigmented Iba1^+^-melanomacrophages were present within the cutaneous and nodal tumor.

#### HRP-Pig

The biopsy of the skin (Additional Figure A4a-c) showed within the superficial dermis and extending into the subcutis parts of a cell-rich, highly pigmented neoplastic mass with reduced degree of pigmentation towards the subcutis. Neoplastic cells were arranged in sheets supported by minimal fibrovascular stroma. The cells were medium-sized to large, with either round to polygonal or spindeloid shape, scant to moderate amount of eosinophilic, granular cytoplasm that contained variable amounts of brown-black, granular pigment that discolored after bleaching (melanin) and indistinct cell borders. They contained a centrally located, round to oval, medium-sized nucleus with finely stippled chromatin and up to two prominent basophilic nucleoli. There was moderate anisocytosis and anisokaryosis and a mitotic rate of 3 mitoses per 2.37 mm^2^.

The biopsy of the subiliac lymph node showed an infiltration of a moderately cellular, highly pigmented neoplastic mass replacing nodular architecture and few remaining follicular aggregates of lymphocytes. Neoplastic cells were arranged in sheets supported by minimal fibrovascular stroma. The cells were medium-sized to large, with either round to polygonal or spindeloid shape, scant to moderate amount of eosinophilic, granular cytoplasm that contained variable amounts of brown-black, granular pigment that discolored after bleaching (melanin) and indistinct cell borders. They contained a centrally located, round to oval, medium-sized nucleus with finely stippled chromatin and up to two basophilic nucleoli. There was moderate anisocytosis and anisokaryosis and a mitotic rate of 0 mitoses per 2.37 mm^2^.

Immunohistochemically, moderate numbers of cutaneous and nodal tumor cells showed positive immunolabelling for MelanA and PNL2. Additionally, highly pigmented Iba1^+^-melanomacrophages were present within the cutaneous and nodal tumor.

### Radiological examination and computed tomography (CT)

#### General information

The radiological examination protocol and computed tomography protocol are described in the Additional File 1, A1.4 and A1.5, respectively.

#### Red-brown DuC-pig

Radiographic examination revealed a diffuse, symmetrical, unstructured interstitial lung pattern with centrally accentuated areas of alveolar lung pattern.

CT findings were compatible with radiographic findings and revealed an unstructured interstitial lung pattern with areas of soft tissue attenuation and the suspicion of mild associated lymphadenopathy.

#### Black DuC-pig

Radiographic examination (Fig. 4a-c) revealed a ventrally accentuated alveolar lung pattern, and a dorsally accentuated interstitial lung pattern with nodular like soft tissue lesions. The findings were more pronounced compared to the red-brown DuC-pig. A cutaneous soft tissue lesion was noted at the right thoracic wall at the level of the 11th to 13th rib, compatible with the clinical examination of the patient. There was suspicion of mild thoracic effusion.

Computed tomography examination (Fig. 5a-b) revealed multiple cutaneous and intermuscular cavitary-like mass lesions, a severe increased attenuation of the lung parenchyma with areas of soft tissue attenuation and nodular-like increased attenuation in the dorsal aspects of the lung. There was evidence for mild thoracic effusion, moderate lymphadenopathy, multifocal nodular hepatopathy, mild abdominal effusion and peritoneal thickening.

#### DuP-pig

The radiographic examination of the thorax revealed no abnormalities.

#### HRP-pig

Radiographic examination revealed a diffuse, symmetrical, unstructured interstitial lung pattern with emphasis on the central areas of the lung. In these areas, a mixed interstitial to alveolar lung pattern was detected.

CT findings were compatible with radiographic findings and revealed a generalized unstructured, partly nodular increased attenuation of the lung parenchyma with peripheral soft tissue opaque fibrous bands. There was no evidence of lymphadenopathy or thoracic effusion.

A large, moderately heterogeneous and cavitary, well-defined, ovoid, soft tissue opaque subcutaneous mass lesion was identified at the level of the right abdominal wall, causing a moderate mass effect and lateral deviation of the abdominal organs, but no invasion into the abdominal cavity. Similar apparent, but smaller lesions were present at the left abdominal wall and right lateral to the third lumbar transverse vertebra. The right medial iliac lymph nodes were moderately enlarged.

### Broncho-alveolar lavage

A broncho-alveolar lavage (BAL) according to Ganter and Hensel [20] was performed in all four pigs under general anesthesia (ketaminhydrochlorid 20 mg/kg body weight intramuscularly [Ketamin 100 mg/ml, cp. pharma, Burgdorf, Germany], azaperone 2 mg/kg body weight intramuscularly [Sedanol 40 mg/ml, WDT, Garbsen, Germany]) with the aim to detect lung metastases. The BAL-fluid (BALF) was analyzed cytologically. Indication of lung metastases was solely found in the black DuC-pig: A black colored sediment could be seen macroscopically after centrifugation of the BALF. Cytology of the BALF revealed numerous cells with variable amounts of brown-black granules (melanin): Two cell populations were identified, one presumably melanocytes, the other one presumably melanophages (Fig. 3).

## Discussion and conclusions

The cases presented herein serve as illustrative examples showcasing the diverse manifestations of this neoplastic disease. Lethal courses of the disease with extensive metastases into several internal organs early in life are described [13]. However, regression within one year after birth is the most common course (approximately 95%) of disease in piglets affected by congenital melanocytic lesions. Congenital melanomas in horses show a similar behavior and are also usually benign tumors [3]. Nevertheless, the course of this neoplastic disease is unpredictable in pigs. While several prognostic factors for prediction of clinical behavior of melanocytic neoplasms are established for other domestic species (e.g. dogs: tumor location, tumor size, ulceration, mitotic count, nuclear atypia, degree of pigmentation, level of infiltration, vascular invasion, lymph node metastases) [21], these do not exist for pigs. For example, the mitotic count with a cutoff value of 3 mitoses per 10 high power fields/2.37 mm^2^ is a key prognostic indicator for melanocytic tumors in dogs. In addition, lymph node metastases are indicative for malignancy. In our cases, two pigs showed lymph node metastases and a variable degree of pigmentation with partly amelanotic areas, but the primary tumor went into regression in both pigs. Moreover, cutaneous melanomas of two pigs had a mitotic count of ≥ 3 mitotic figures per 2.37 mm^2^, but the courses of disease were clearly different (fatal outcome versus regression of primary tumor). In addition, immunohistochemistry revealed metastasis in the inguinal lymph node of the DuP-pig, although a mitotic count of 0 mitotic figures per 2.37 mm^2^ was detected in the biopsy of the primary tumor. The low mitotic rate could be explained by the fact that only a biopsy was taken for histopathological examination, meaning that not necessarily the most mitotic areas were available for histologic examination. This is also reflected in the comparison of the mitotic rates of the biopsy and the whole primary tumor of the black DuC. One mitosis per 2.37 mm² was detected in the biopsy, while a mitotic rate of 12 mitoses per 2.37 mm² was observed in the entire tumor. Overall, the tumors of three presented pigs displayed features of malignancy described for canine melanocytic neoplasia. Only one pig showed a malignant clinical course, whereas the primary tumor of the two other pigs went into regression, and both pigs showed additional signs of regression in form of leukoderma and leukotrichia. Accordingly, applied prognostic factors for canine melanocytic tumors were not suitable for prediction of disease progression in our pig cases. These findings underline why a standardized histopathological classification into benign melanocytoma and malignant melanoma in pigs is not possible. Immunological factors may have a greater impact on the survival of this neoplastic disease in pigs than, for example, the aggressiveness of the primary tumor. Leukoderma, leukotrichia and discoloration of the iris are clinical signs that frequently occur during tumor regression of porcine cutaneous melanocytic neoplasia [15, 17, 22]. Leukoderma is also reported in a small proportion of human patients with malignant melanoma [23] and can be accompanied by spontaneous regression of this neoplastic disease [24]. It is assumed that an immune reaction of the body is responsible for this clinical sign [25]. The immune reaction seems to be directed against antigens of the neoplastic melanoma cells, which are shared with non-neoplastic melanocytes. Furthermore, human patients that undergo immune therapy against metastatic malignant melanoma can develop leukoderma and leukotrichia [26, 27]. These clinical signs are associated with a favorable prognosis in humans both as spontaneous clinical sign of malignant melanoma [24] and as occurring clinical sign during immune therapy [28, 29]. Black pigmentation of lymph nodes that was a sign of metastases in our cases, can also indicate melanosis caused by melanophages during tumor regression [30]. Melanosis can be seen as an expression of tumor degradation and removal of melanin by macrophages. In general, the histopathological course of regression of cutaneous melanocytic neoplasia in pigs is described as a biphasic process [31]: In the first phase, an infiltration of large numbers of melanophages is observable, while in the second phase a lymphocytic infiltration of the residual tumor is present. The histopathological differentiation of melanocytes and melanophages can also be difficult in native histological specimens. However, it is possible to reliably distinguish porcine melanocytes from porcine melanophages using immunohistochemical staining, e.g. with specific markers for melanocytes such as MelanA and PNL2 or the specific macrophage marker ionized calcium-binding adaptor molecule 1 (Iba 1) [30].

It is noteworthy that the clinical examination of the respiratory tract of the black Duroc crossbred piglet on the day of euthanasia did not reveal any findings despite the severe lesions in the lung. Although the general condition was slightly impaired for the first time on the day of euthanasia, the piglet still had a good feed intake. The cytological examination of the BALF that was conducted previously revealed a high number of melanocytes and melanophages and supported the suspicion of lung metastases of a malignant melanoma that were later confirmed by radiographs, computed tomography and necropsy. In human medicine, cellular analysis of BALF is used to diagnose malignant neoplastic diseases in the pulmonary parenchyma and there are few reports dealing with the use of BALF in connection with the diagnosis of malignant melanoma [32, 33]. Our results show that BAL can be used as a relatively low-cost and easy-to-perform diagnostic tool in pigs to detect neoplastic changes in the lung. To the best of the author’s knowledge, this also marks the first documentation of BAL being utilized in the diagnosis of a metastatic melanoma in pigs. A potential differential diagnosis for the presence of pigmented cells in the BALF in pigs is congenital visceral lung melanosis. However, the pathological examination showed no evidence of this condition.

Exophytic, nodular and macule-like types of cutaneous melanocytic lesions are described [1, 13, 22] and all forms were evident in our cases. The nodular tumor of the Husum Red Pied flattened over time and its surface became uneven. This was seen as a first clinical sign for regression. A macule-like melanocytic lesions was seen in the purebred Duroc and it showed a similar phenotype to other case reports [1, 17, 22, 34]. Vincent-Naulleau et al. [34] and Horak et al. [17] described a complete flattening to a macule-like lesion of former nodular melanoma during regression. Therefore, the macule-like phenotype of the cutaneous lesion could present a final stage of regression.

The heritability of the congenital form in pigs is proven for Duroc breed [1, 11, 35] and the three miniature pig breeds [15, 16, 36, 37]. The genetic basis of congenital melanoma in the Duroc breed is not fully understood [11]. No information is available on possible heredity and the associated inheritance in Husum Red Pied pigs, as this is the first description of a congenital melanoma in this breed. The Husum Red Pied was originally bred at the end of the 19th century period [38]: Ancestors of this breed were the Angeln Saddleback (today also a variety of the breed German Saddleback), Tamworth and other regional North German and Danish pig breeds. The breed disappeared in the late 1960s and the herd book was closed. During the 1980s, pigs exhibiting this phenotype were rediscovered and subsequently subjected to professional breeding efforts aimed at preserving this characteristic. Therefore, it is conceivable that uncontrolled crossbreeding with similar colored Duroc pigs led to manifestation of the hereditary trait that is responsible for the occurrence of congenital melanocytic neoplasia. The Husum Red Pied breed is endangered and population number of breeding stock was only 129 (90 sows and 39 boars) in 2021 [39]. In a population characterized by a limited gene pool, some level of inbreeding is inevitable, even with a carefully designed breeding program. Therefore, if this disease is also hereditary in the Husum Red Pied and not factored into the breeding program, there is a significant risk of an escalating proportion of pigs being born with congenital melanocytic neoplasia within the population.

Cutaneous melanocytic lesions in Duroc were first scientifically reported by Pickens [5] and there are several case reports from the 20th century [8, 11, 35, 40, 41]. The incidence of cutaneous melanocytic lesions in pigs can be considered as low. Several reports of analyzed lesions of slaughter pigs and from pathology laboratories found limited numbers of cutaneous melanocytic lesions [9, 42–47]. The Duroc breed is very frequently used in Germany to produce Duroc-sired fattening pigs. Duroc genetics are mainly used for artificial insemination and the semen is supplied to the farmer by professional breeding companies. The lack of case reports in recent decades may suggest a decline in this neoplastic disease within professional breeding. However, only recent slaughterhouse surveys on a regional base could provide information on whether the prevalence of this disease has actually decreased. Therefore, it is interesting that two cases of cutaneous melanocytic neoplasia of Duroc pigs from small-scale farms of the same region were presented concurrently to our clinic. It was not possible to find an epidemiological or family link between the two affected farms. The fact that cases of congenital melanocytic neoplasia were observed before on farm DuP, but the affected gilt itself came from a third farm, is an indication of a relatively high incidence of congenital melanocytic neoplasia in small-scale farms in this region. The anamnesis of affected farms revealed that breeding stock (gilts or boars) was bought from other small-scale farms and not from professional breeding companies. Hence, it is conceivable that this practice has led to this hereditary neoplastic disease persisting in an isolated gene pool in small-scale farms of this region, as it was not subject to professional genetic improvement in the past. There is a risk that the incidence of the disease will increase in the affected herds without targeted breeding programs. This highlights the need to raise awareness for this disease among farmers. Affected small scale farms should be advised to consistently exclude affected pigs from further breeding activities. There is a high probability that the purchased boar of farm DuC was the cause of the hereditary disease in this farm, as the pedigree of the breeding sow was fully documented and no cases of congenital melanoma had previously been reported. In this case, only the culling of the boar was recommended.

## Electronic supplementary material

Below is the link to the electronic supplementary material.

Supplementary Material 1

Supplementary Material 2

Supplementary Material 3

Supplementary Material 4

Supplementary Material 5

## Data Availability

No datasets were generated or analysed during the current study.

## Abbreviations

BAL: Broncho–alveolar lavage
BALF: Broncho–alveolar lavage fluid
cm: centimeter
DuC: Duroc crossbred
DuP: Duroc purebred
HRP: Husum Red Pied
Iba 1: Ionized calcium–binding adaptor molecule 1
Ln.: Lymph node
Lnn.: Lymph nodes
G/l: Giga per litre
MeLiM: Melanoblastoma bearing Libechov Miniature Pig
