# Supplementary figures and images for: Simultaneous occurrence of cutaneous melanocytic neoplasia in Duroc and German Saddleback pigs from three smallholder farms
Source: BMC Vet Res. 2025 Jun 27;21:409. doi: 10.1186/s12917-025-04863-0 (PMC12203717; doi:10.1186/s12917-025-04863-0)

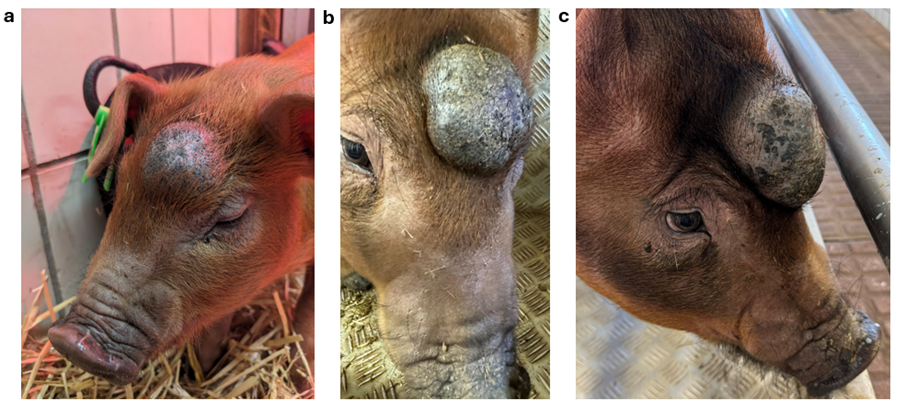

Supplement: Supplementary file 1 — Supplementary Material 1 [file 12917_2025_4863_MOESM1_ESM.png]

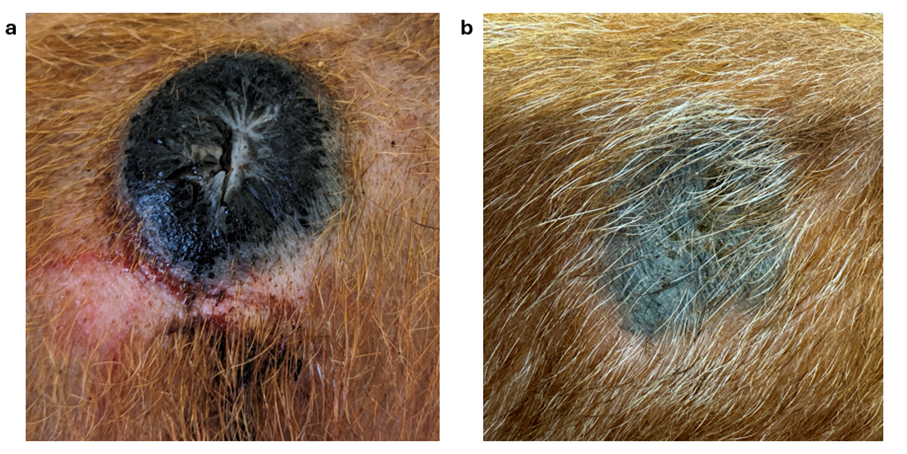

Supplement: Supplementary file 2 — Supplementary Material 2 [file 12917_2025_4863_MOESM2_ESM.png]

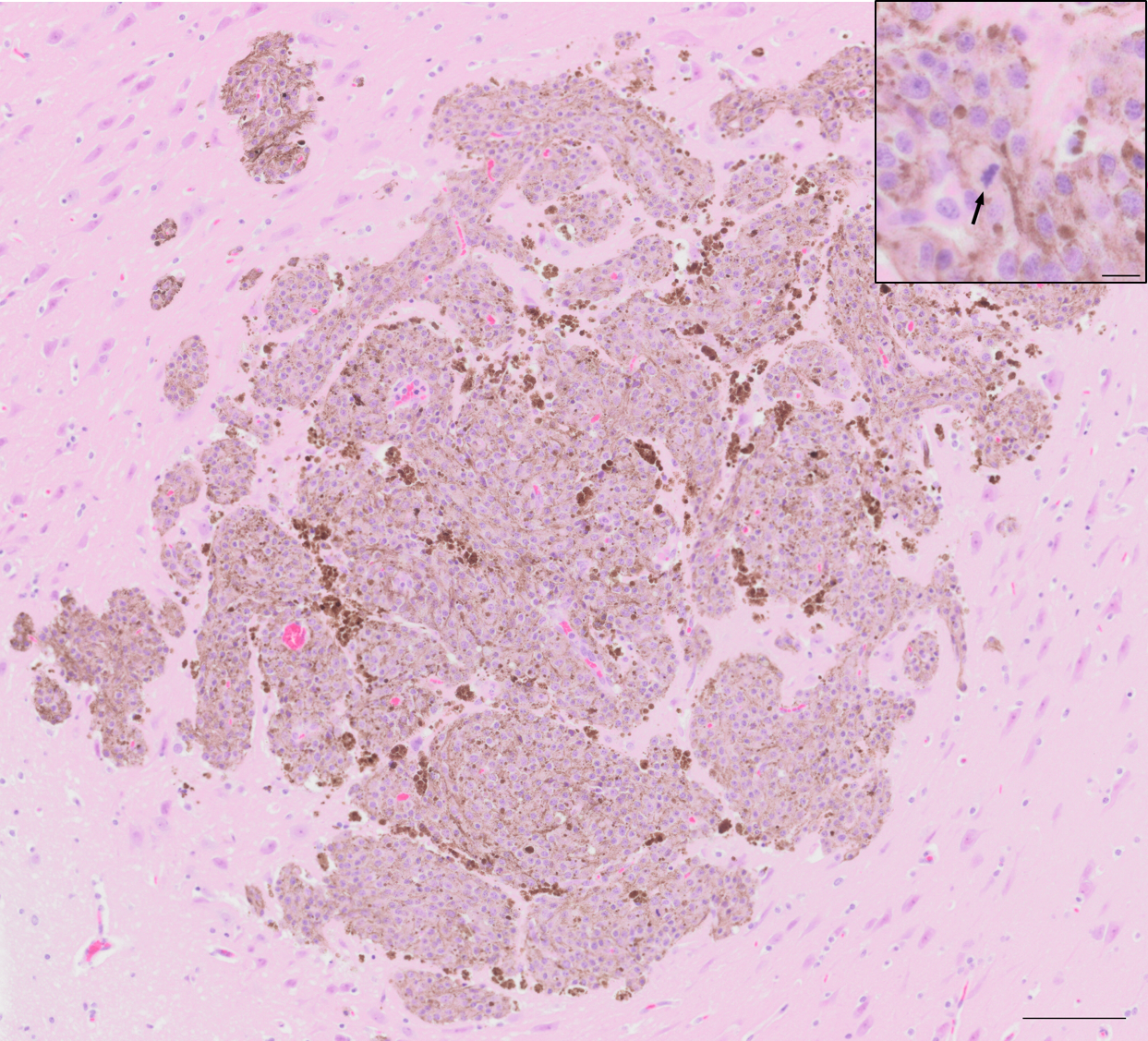

Supplement: Supplementary file 3 — Supplementary Material 3 [file 12917_2025_4863_MOESM3_ESM.tif]

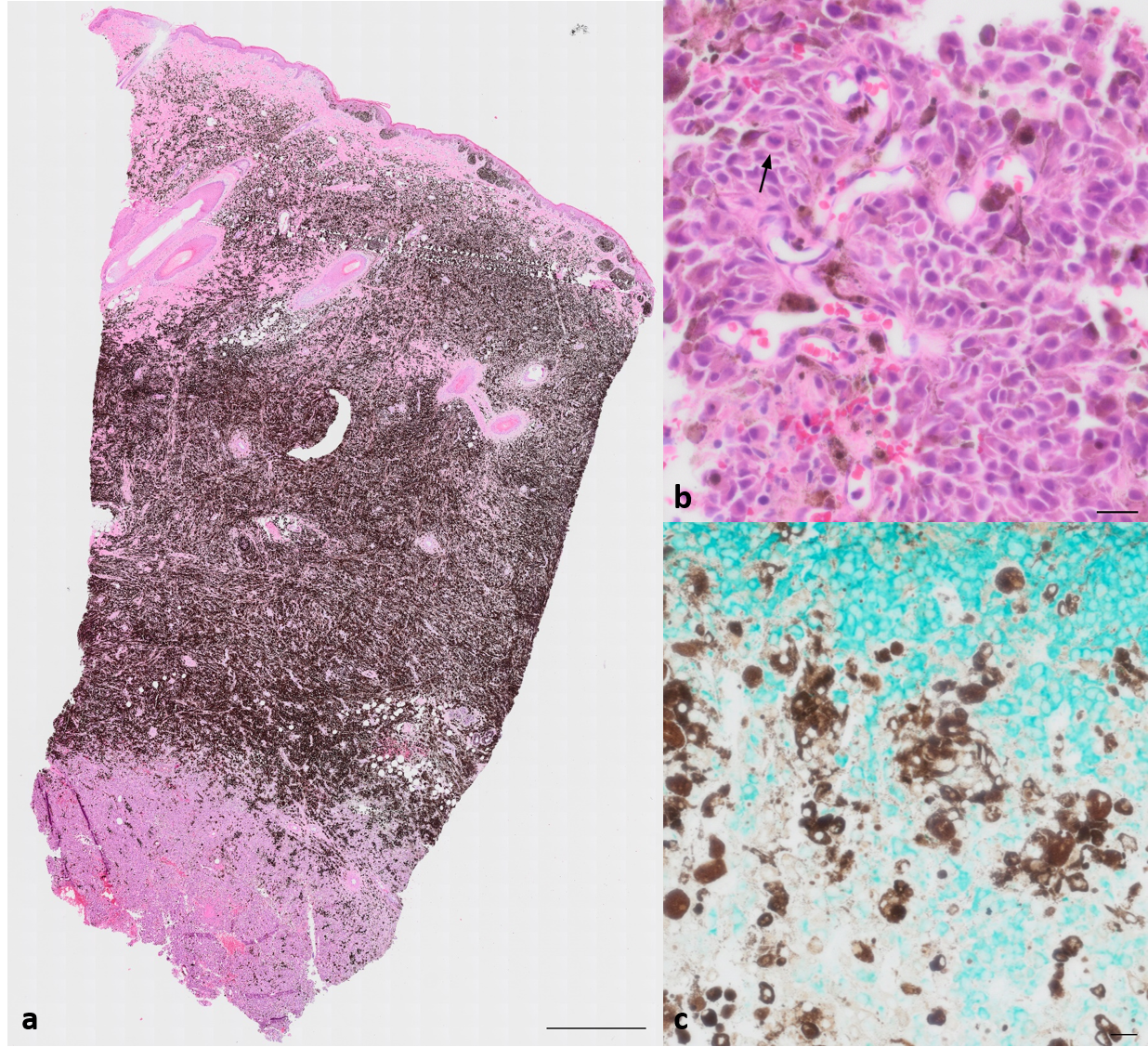

Supplement: Supplementary file 4 — Supplementary Material 4 [file 12917_2025_4863_MOESM4_ESM.tif]
