# Supplementary material for: Simultaneous occurrence of cutaneous melanocytic neoplasia in Duroc and German Saddleback pigs from three smallholder farms
Source: BMC Vet Res. 2025 Jun 27;21:409. doi: 10.1186/s12917-025-04863-0 (PMC12203717; doi:10.1186/s12917-025-04863-0)
Supplement: Supplementary file 5 — Supplementary Material 5 [file 12917_2025_4863_MOESM5_ESM.docx]

**Additional file 1:**

**Simultaneous occurrence of cutaneous melanocytic neoplasia in Duroc and German Saddleback pigs from three smallholder farms**

Thies J. Nicolaisen^1,a, *^, Melanie Stoff ^2^, Manon Mikic^3^, Anna Lena Maschmeier^2^, Johannes Buchallik-Schregel^1^, Alexandra von Altrock^1^, Maybrit Wirtz^3^, Saskia Neubert^1^, Andreas Beineke^2^, Doris Höltig^1^, Isabel Hennig-Pauka^4^, Martin Ganter^1^

^1^ Clinic for Swine and Small Ruminants, Forensic Medicine and Ambulatory Clinic, University of Veterinary Medicine Hannover, Foundation, Hannover, Germany

^2^ Department of Pathology, University of Veterinary Medicine Hannover, Foundation, Hannover, Germany

^3^ Department of Small Animal Medicine and Surgery, University of Veterinary Medicine Hannover, Foundation, Hannover, Germany

^4^ Field Station for Epidemiology (Bakum), University of Veterinary Medicine Hannover, Foundation, Hannover, Germany

^a^ current address: Institute for Animal Hygiene, Animal Welfare and Farm Animal Behaviour, University of Veterinary Medicine Hannover, Foundation, Germany

^*^ Corresponding author: [thies.jesper.nicolaisen@tiho-hannover.de](mailto:thies.jesper.nicolaisen@tiho-hannover.de)

**A.1 Clinical examination and diagnostics:**

**A1.1 Clinical examination:**

**Table A1.1.1 Results of the clinical examination of the three pigs (red-brown DuC-pig, DuP-pig, HRP-pig) at hospitalization**

| Pig and date of examination |  | DuC red-brown, 27.11.2023 | DuP pig, 09.01.2024 | HRP-pig, 11.12.2023 |
| --- | --- | --- | --- | --- |
| Adspection | Body posture | Equal weight-bearing on all four limbs, straight back line, head held high | Equal weight-bearing on all four limbs, straight back line, head held high | Equal weight-bearing on all four limbs, straight back line, head held high |
|  | Behavior | Calm and alert | Calm and alert | Calm and alert |
|  | Nose | Straight, symmetrical, snout moist, no discharge / deposits | Straight, symmetrical, snout moist, no discharge / deposits | Straight, symmetrical, snout moist, no discharge / deposits |
|  | Eyes | Clear on both sides, without discharge | Clear on both sides without discharge | Clear on both sides without discharge |
|  | Ears | Ear veins not congested, small amounts of cerumen in ear canal | Ear veins not congested, no cerumen in ear canal | Ear veins not congested, no cerumen in ear canal |
|  | Respiratory Rate | 44 breathings / minute | 32 breathings / minute | 30 breathings / minute |
|  | Respiratory Type | Costo-abdominal | Costo-abdominal | Costo-abdominal |
|  | Feces | Not assessable during clinical examination | Feces formed | Not assessable during clinical examination |
|  | Urine | Not assessable during clinical examination | Clear urine without impurities | Not assessable during clinical examination |
|  | Movement | No lameness | No lameness | No lameness |
|  | Skin | center of the forehead: a growth with a diameter of 4 cm, approx. 1-2 cm high: black, hairless, dry; small number of  bristles at the edge of the growth | Left flank: suspicion of melanoma with a diameter of approx. 3 cm | Right flank: dorsal growth (diameter 3-4 cm, height 1 cm) near the lumbar vertebrae: black, dry, no signs of inflammation |
|  | Bristles | No abnormalities | No abnormalities | No abnormalities |
| Auscultation | Heart | Frequency; 152 beats per minute, Intensity: regular and strong; Rhythm: regular; Separability of heart sounds: Good separability; heart murmurs: No | Frequency: 72 beats per minute; Intensity: regular and strong; Rhythm: regular; Separability of heart sounds: Good separability; heart murmurs: No | Frequency: 124 beats per minute; Intensity: regular and strong; Rhythm: regular; Separability of heart sounds: Good separability; heart murmurs: No |
|  | Lung (Upper, medium and low lung border) | All lung borders: low-grade inspiratory breath sound | Upper and medium lung borders: low-grade inspiratory breath sound; lower lung borders: no breath sounds | All lung borders: low-grade inspiratory breath sound |
| Palpation | Rectal temperature | 38.0°C | 38.0°C | 39.7°C |
|  | Body condition score (1-5) | 3 | 3 | 3 |
|  | Pulse | Not palpable | Not palpable | Not palpable |
|  | Body surface temperature | Uniform body surface temperature, decreasing towards the limbs | Uniform body surface temperature, decreasing towards the limbs | Uniform body surface temperature, decreasing towards the limbs |
|  | Conjunctiva and episcleral vessels | Conjunctiva: red; Fine pattern of episcleral vessels | Conjunctiva: red; Fine pattern of episcleral vessels | Conjunctiva: red; Fine vessels of episcleral vessels |
|  | Lymphonodi inguinales superficiales | Left: firm-elastic, moveable under the skin, no increased warmth, not indicative of pain  Right: firm-elastic, movable under the skin, no increased warmth, not indicative of pain | Left: 10 x 5 x 5 cm, firm-elastic, movable under the skin, no increased warmth, not indicative of pain.  Right: 2 x 2 x 4 cm, firm-elastic, movable under the skin, no increased warmth, not indicative of pain | Left: 1cm diameter, firm-elastic, movable under the skin, no increased warmth, not indicative of pain.  Right: diameter 1 cm, firm-elastic, movable under the skin, no increased warmth, not indicative of pain |
|  | Other lymph nodes | - | - | Lymphonodus subiliacus (right): severe enlargement: 5 x 2 x 2 cm, firm-elastic, movable under the skin, no increased warmth, not indicative of pain |
|  | Coughing | Coughing triggerable (one coughing bout) | Coughing not triggerable | Coughing not triggerable |
|  | Abdominal wall | Soft, not indicative of pain | Soft, not indicative of pain | Soft, not indicative of pain |
|  | Udder / testicles | juvenile | juvenile | juvenile |
|  | Joints and Claws | Joints: No signs of inflammation; Claws: Normal shape, normal abrasion | Joints: No signs of inflammation; Claws: Normal shape, normal abrasion | Joints: No signs of inflammation; Claws: Normal shape, normal abrasion |
|  | others |  |  | Several black pigmented skin areas with a diameter between 0.5-1.0 cm in the following locations: left scapula, mammary gland and left metatarsus; Tail deformity (kinked tail) in the last third |

**Table A1.1.2 Results of the clinical examinations of the DuC black pig at hospitalization (27.11.2023), euthanasia (14.12.2023) and one time-point in between (08.12.2023)**

| Date of clinical examination |  | 27.11.2023 | 08.12.2023 | 14.12.2023 |
| --- | --- | --- | --- | --- |
| Adspection | Body posture | Equal weight-bearing on all four limbs, low-grade kyphosis, head held high | Equal weight-bearing on all four limbs, straight back line, head held high | Equal weight-bearing on all four limbs, straight back line, head held high |
|  | Behavior | Calm and alert | Calm and alert | Moderately impaired general condition: No exploratory behavior displayed when examiner entered the pen; pig separates itself from conspecific and lies down in resting area |
|  | Nose | Straight, symmetrical, snout moist, no discharge / deposits | Straight, symmetrical, snout moist, no discharge / deposits | Straight, symmetrical, snout moist, no discharge / deposits |
|  | Eyes | Clear on both sides without discharge | Clear on both sides without discharge | Clear on both sides without discharge; inner eyelid of right eye with lentil-sized melanoma |
|  | Ears | Ear veins not congested, small amounts of cerumen in ear canal | Ear veins not congested, small amounts of cerumen in ear canal | Ear veins not congested, No cerumen in ear canal |
|  | Respiratory Rate | 24 breathings / minute | 24 breathings / minute | 24 breathings / minute |
|  | Respiratory Type | Costo-abdominal | Costo-abdominal | abdominal |
|  | Feces | Not assessable during clinical examination | Formed feces | diarrhea |
|  | Urine | Not assessable | Clear urine without impurities | Not assessable |
|  | Movement | No lameness | No lameness | No lameness |
|  | Skin | Right abdominal wall: 6 x 4 cm large, fissured growth: black, encrusted, bloody and oozing.  Right thigh: 3x1 cm skin wound, healing. | Right abdominal wall: 6 x 4 cm large, fissured growth: black, encrusted, bloody and oozing. | Right abdominal wall: 6 x 4 cm large, fissured growth: black, encrusted, bloody and oozing. |
|  | Bristles | No abnormalities | No abnormalities | Dull, matte and overlong bristles |
| Auscultation | Heart | Frequency: 120 beats per minute; Intensity: regular and strong; Rhythm: regular rhythm; Separability of heart sounds: Good separability; Heart murmurs: No | Frequency: 120 beats per minute; Intensity: regular and strong; Rhythm: regular rhythm; Separability of heart sounds: Good separability; Heart murmurs: No | Frequency: 128 beats per minute; Intensity: regular and strong; Rhythm: regular rhythm; Separability of heart sounds: Good separability; Heart murmurs: No |
|  | Lung (Upper, medium and low lung borders) | All lung borders: Low-grade inspiratory breath sound | All lung borders: No breath sounds | Upper lung borders: Low-grade inspiratory breath sound; medium and low lung borders: No breath sounds |
| Palpation | Rectal temperature | 38.5°C | 38.7° C | 40.0 °C |
|  | Body condition score (1-5) | 2 | 2.5 | 2.5 |
|  | Pulse | Not palpable | Not palpable | Not palpable |
|  | Body surface temperature | Uniform body surface temperature, decreasing towards the limbs | Uniform body surface temperature, decreasing towards limbs | Uniform body surface temperature, decreasing towards limbs |
|  | Conjunctiva and episcleral vessels | Conjunctiva: red; slightly permeable episcleral vessels | Conjunctiva: pink; fine pattern of episcleral vessels | Conjunctiva: Pale pink; fine pattern of episcleral vessels |
|  | Lymphonodi inguinales superficiales | Right: 2x2 cm, firm-elastic, moveable under the skin, no increased warmth, not indicative of pain.  Left: 1x1 cm, firm-elastic, movable under the skin, no increased warmth, not indicative for pain | Right: 2x2 cm, firm-elastic, moveable under the skin, no increased warmth, not indicative of pain.  Left: 1x1 cm, firm-elastic, moveable under the skin, no increased warmth, not indicative for pain | Right: 2x2 cm, firm-elastic, moveable under the skin, no increased warmth, not indicative of pain.  Left: 1x1 cm, firm-elastic, moveable under the skin, no increased warmth, not indicative for pain |
|  | Other lymph nodes | Lymphonodus subiliacus (right): size: 4x3x1.5 cm, firm, movable under the skin, not indicative of pain | Lymphonodus subiliacus (right): 4x3x1.5 cm, firm-elastic, movable under the skin, no increased warmth, not indicative of pain | Lymphonodus subiliacus (right): 4x3x1.5 cm, firm-elastic, movable under the skin, no increased warmth, not indicative of pain |
|  | Coughing | Coughing not triggerable | Coughing not triggerable | Coughing not triggerable |
|  | Abdominal wall | Soft, not indicative of pain | Soft, not indicative of pain | Soft, not indicative of pain |
|  | Udder / testicles | juvenile | juvenile | juvenile |
|  | Joints and claws | Joints: No signs of inflammation; Claws: normal shape, normal abrasion | Joints: No signs of inflammation; Claws: Normal shape, normal abrasion | Joints: No signs of inflammation; Claws: Normal shape, normal abrasion |

**A 1.2. Rectal temperature**

**A1.2.1 Rectal temperature (°C) of the black DuC-Pig measured in the morning and evening from 27.11.2023 (hospitalization) to 14.12.2023 (euthanasia)**

| Date |  | 27.11.23 | 28.11.23 | 29.11.23 | 30.11.23 | 01.12.23 | 02.12.23 | 03.12.23 | 04.12.23 | 05.12.23 | 06.12.23 | 07.12.23 | 08.12.23 | 09.12.23 | 10.12.23 | 11.12.23 | 12.12.23 | 13.12.23 | 14.12.23 |
| --- | --- | --- | --- | --- | --- | --- | --- | --- | --- | --- | --- | --- | --- | --- | --- | --- | --- | --- | --- |
| Time | Morning | - | 39.0 | - | 38.5 | 39.2 | 39.0 | 38.1 | - | - | - | - | - | 38.7 | 38.7 | - | 38.9 | - | 40.0 |
|  | Evening | 39.2 | 38.5 | 38.1 | 38.9 | 38.5 | - | 39.3 | 37.7 | 38.8 | 39.0 | 38.9 | 38.7 | - | - | 37.6 | 38.0 | 39.4 | - |

**A1.3 Hematological examination:**

Sampling location:

Blood was collected from the vena cava cranialis in DuC red-brown pig, DuC black pig and HRP-pig and from vena jugularis externa in DuP-pig. The sampling system consisted of a EDTA S-Monovette® [Sarstedt, Nümbrecht, Germany] and a Sterican® needle 18G, 1.2 mm x 40 mm [Braun, Melsungen, Germany]).

**Table A1.3.1 Complete blood count of the four pigs (DuC black pig, DuC red-brown pig, DuP-pig, HRP-pig) on the day of hospitalization**

| **Parameter** | **Unit** | **Reference range** | **DuC black pig, 27.11.2023** | **DuC red-brown pig, 27.11.2023** | **DuP-pig, 09.01.2024** | **HRP-pig, 12.12.2023** |
| --- | --- | --- | --- | --- | --- | --- |
| Leukocytes | Giga / liter | 10-20 | 20.9 | 19.2 | 14.9 | 16.3 |
| Corrected leukocytes | Giga / liter | 10-22 | 20.8 | 19.2 | 14.9 | 16.3 |
| Erythrocytes | Terra / liter | 5.8-8.2 | 6.58 | 7.48 | 6.8 | 6.31 |
| Hemoglobin | Gram / liter | 108-148 | 109 | 121 | 125 | 112 |
| Hematocrit | Liter / liter | 0.33-0.45 | 0.33 | 0.36 | 0.37 | 0.34 |
| Mean corpuscular volume | Femtoliters | 50-85 | 50.2 | 48.1 | 54.4 | 53.9 |
| Mean corpuscular hemoglobin | Picogram | 17-21 | 16.6 | 16.2 | 18.4 | 17.7 |
| Mean corpuscular hemoglobin concentration | Gram / liter | 300-350 | 330 | 336 | 338 | 329 |
| Thrombocytes | Giga / liter | 180-600 | 625 | 366 | 68 * | 465 |
| Lymphocytes | Percent |  | 15 | 86 | 38 | 34 |
| Segmented neutrophils | Percent |  | 77 | 11 | 56.5 | 53.5 |
| Band neutrophils | Percent |  | 5.5 | 0 | 5 | 6 |
| Metamyelocytes | Percent |  | 0 | 0 | 0 | 0.5 |
| Myelocytes | Percent |  | 0 | 0 | 0 | 0 |
| Eosinophilic granulocytes | Percent |  | 0 | 0.5 | 0 | 1 |
| Basophilic granulocytes | Percent |  | 0 | 0 | 0.5 | 0.5 |
| Monocytes | Percent |  | 2.5 | 2.5 | 0 | 4.5 |
| Normoblasts | Percent |  | 0.5 | 0 | 0 | 0 |
| Lymphocytes | Giga / liter | 6.0-16.0 | 3.12 | 16.51 | 5.66 | 5.54 |
| Segmented Neutrophils | Giga / liter | 1.0-8.2 | 16.01 | 2.11 | 8.42 | 8.72 |
| Banded Neutrophils | Giga / liter | 0-1.5 | 1.14 | 0 | 0.74 | 0.98 |
| Metamyelocytes | Giga / liter |  | 0 | 0 | 0 | 0.08 |
| Myelocytes | Giga / liter |  | 0 | 0 | 0 | 0 |
| Eosinophilic granulocytes | Giga / liter | 0-1.3 | 0 | 0.1 | 0 | 0.16 |
| Basophilic granulocytes | Giga / liter | 0-0.5 | 0 | 0 | 0.07 | 0.08 |
| Monocytes | Giga / liter | 0-1.0 | 0.52 | 0.48 | 0 | 0.73 |
| Anisocytosis |  |  | ++ | +++ | + | ++ |
| Polychromasia |  |  | ++ | ++ | + | ++ |
| Poikilocytosis |  |  | ++ | + |  | ++ |

* a blood clot was present in the blood sample

**A1.4 Radiographic examinations:**

Radiographic examinations of the thorax of all patients were performed using a diagnostic X-ray apparatus (Bucky Diagnostic Floor System, Philips), a tabletop technique of 70 to 73 peak kilovoltage (kVp) and 8 milliampere-seconds (mAs), and a 1-m focal-film distance. Images were stored in a picture archiving communication system (PACS) and analyzed by use of a medical image viewer (EasyVet Imaging). Right lateral (RL), left lateral (LL) and ventrodorsal (VD) were obtained.

**A1.5 Computed tomography:**

Computed tomography (CT) imaging of the black DuC-pig, red-brown DuC-pig and HRP-pig was performed with the pigs in sternal recumbency and under general anesthesia using Philips IQon Spectral CT (Philips Healthcare Germany). Scans were acquired with 120-140 kilovolts (kV) maximum tube potential, a pitch of 0,6 with a gantry rotation time of 0,5 s, a slice thickness of 2 mm, and a 512-image matrix. CT scans were performed with standardized protocols for the abdomen and thorax, using lung-, soft tissue- and bone reconstruction algorithms with the appropriate bone (window length: 800, window width: 2000), soft tissue (window length: 60, window width: 350) and lung windows (window length: -600, window width: 1600).

**A 1.6 Anaesthesia protocols**

*1.6.1: Punch biopsies, radiological examination and broncho-alveolar lavage*

Ketaminhydrochlorid 20 mg/kg body weight intramuscularly [Ketamin 100 mg/ml, cp pharma, Burgdorf, Germany], azaperone 2 mg/kg body weight intramuscularly [Sedanol 40 mg/ml, WDT, Garbsen, Germany]

*1.6.2 Radiological examination and computed tomography (CT)*

Induction: Ketaminhydrochlorid 20 mg/kg body weight intramuscularly [Ketamin 100 mg/ml, cp pharma, Burgdorf, Germany], azaperone 2 mg/kg body weight intramuscularly [Sedanol 40 mg/ml, WDT, Garbsen, Germany]. Maintenance of anesthesia with isoflurane (Isofluran Baxter vet 1000 mg/g, Baxter, Unterschleißheim, Germany).

**A1.7 Histopathological examination and immunohistochemistry:**

The biopsies were immersion-fixed with 10% neutral-buffered formalin and embedded in paraffin and sections of 3µm thickness were stained with hematoxylin and eosin. In order to demonstrate the presence of melanin within the specimens, sections were bleached with potassium permanganate followed by staining with hematoxylin and eosin.

The immunohistochemical examination of samples was carried out using the avidin-biotin-peroxidase method (ABC-method; Vectastain®Elite® ABC-HRP Kit, Vector Laboratories) as described (1) and Histogreen (HISTOPRIME HistoGreen, Biozol) was used as chromogen for visualization. Antibodies against MelanA (mouse monoclonal antibody; Dako/Agilent, Clone A103; dilution 1:400) and PNL2 (mouse monoclonal antibody; Santa Cruz, sc-59306; dilution 1:500) were used to detect melanocytic cells, while macrophages were identified using Iba1 (rabbit polyclonal antibody; Fujifilm Wako Pure Chemical Corporation; dilution 1:2000). A negative control was performed for each specimen using ascites fluid from non-immunized Balb/C mice (mouse monoclonal antibodies [MelanA, PNL2]; CL8100, Biologo) and serum from non-immunized rabbits (rabbit polyclonal antibody [Iba1]; R4505; Merck) instead of the primary antibodies. A malignant melanoma of a dog and skin from a pig has been used as positive controls for MelanA and PNL2. Lymph node from a pig was used as positive control for Iba1.


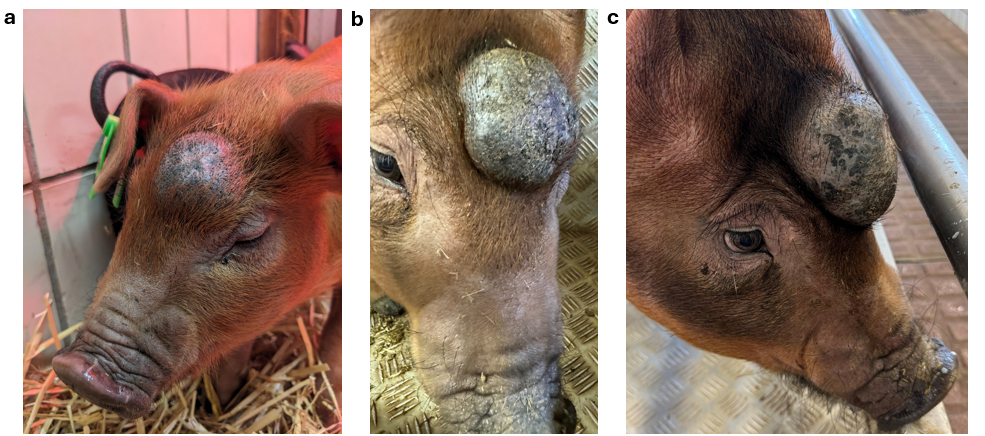

Figure A1a-c: Congenital melanocytic neoplasia on the forehead of the red-brown DuC pig. 1a: 6^th^ weeks of age, 1b: 21^st^ weeks of age; 1c: 29^th^ weeks of age


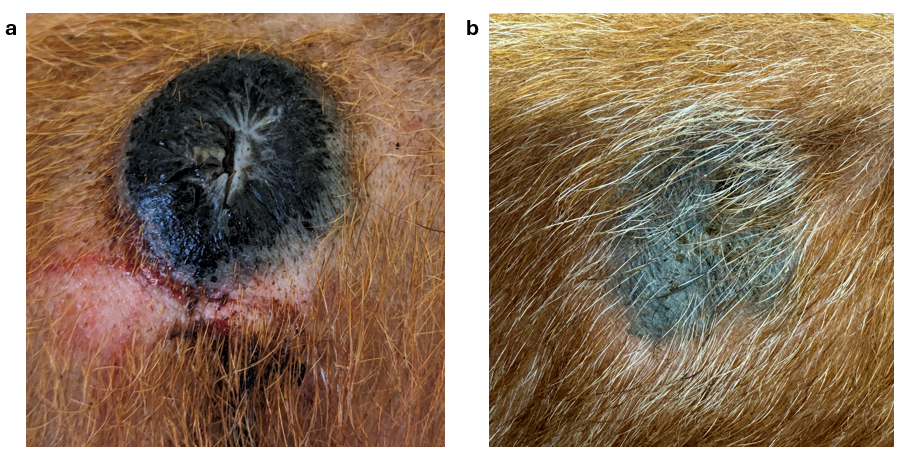


Figure A2a-b: Congenital melanoma in the right flank of the HRP pig at two points in time. 4a: 9^th^ week of age, nodular type of melanoma, black colored with a central amelanotic part. 4b: 26^th^ week of age, flattened type of melanoma with signs of leukotrichia.

**
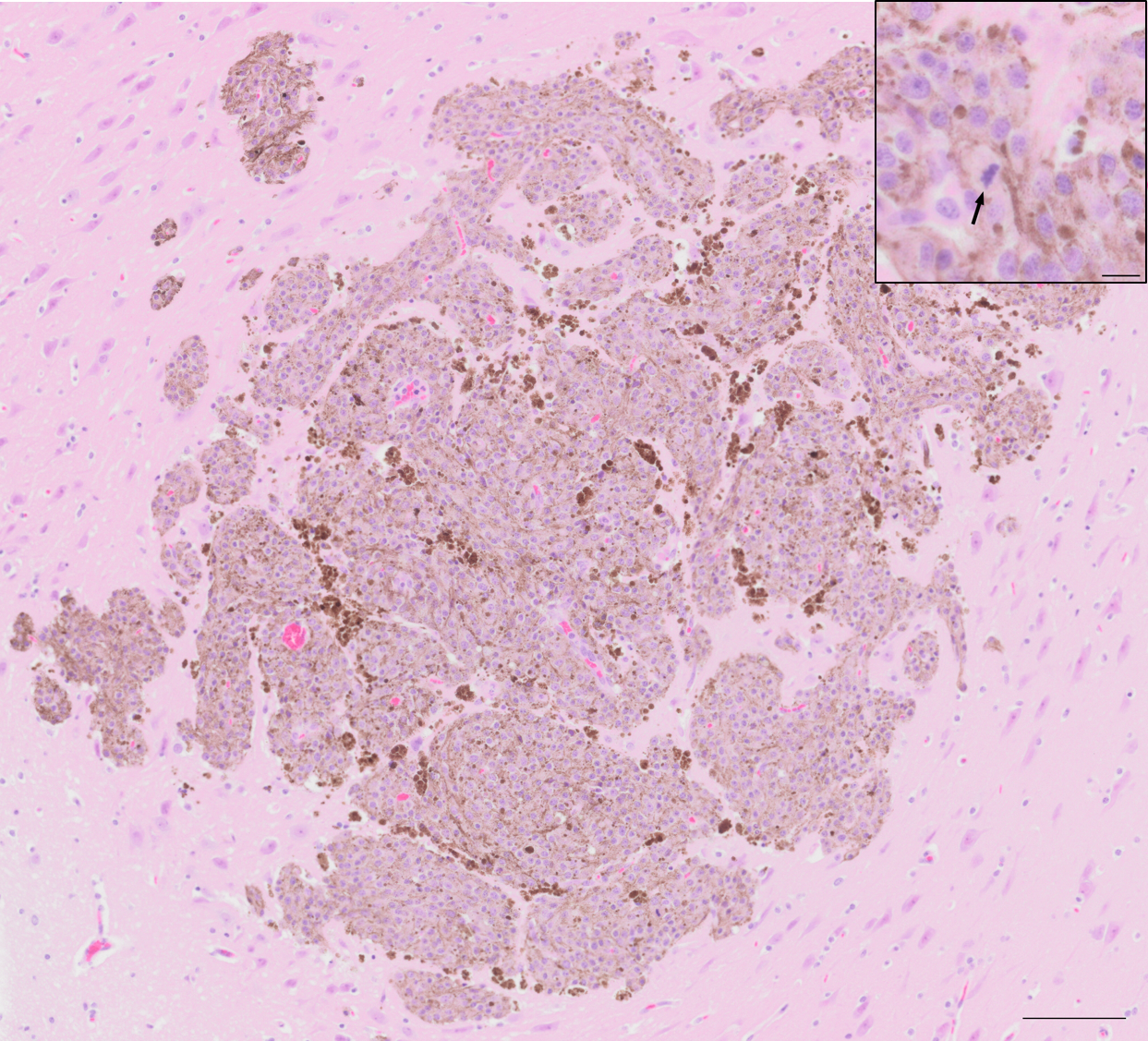
**

Figure A3a-c: Black DuC pig, metastatic melanoma cerebrum. Perivascular accentuated infiltration of variable pigmented tumor cells and melanomacrophages within the cerebral cortex. Hematoxylin and eosin stain, bar = 100 µm. Insert: Neoplastic cells contain a central to paracentral located, round to oval, medium-sized to large nucleus with finely stippled chromatin and up to 2 prominent basophilic nucleoli. There is moderate anisocytosis, anisokaryosis and anisonucleolosis. Occasionally, mitotic figures (arrow) are present. Hematoxylin and eosin stain, scale bar = 10 µm.


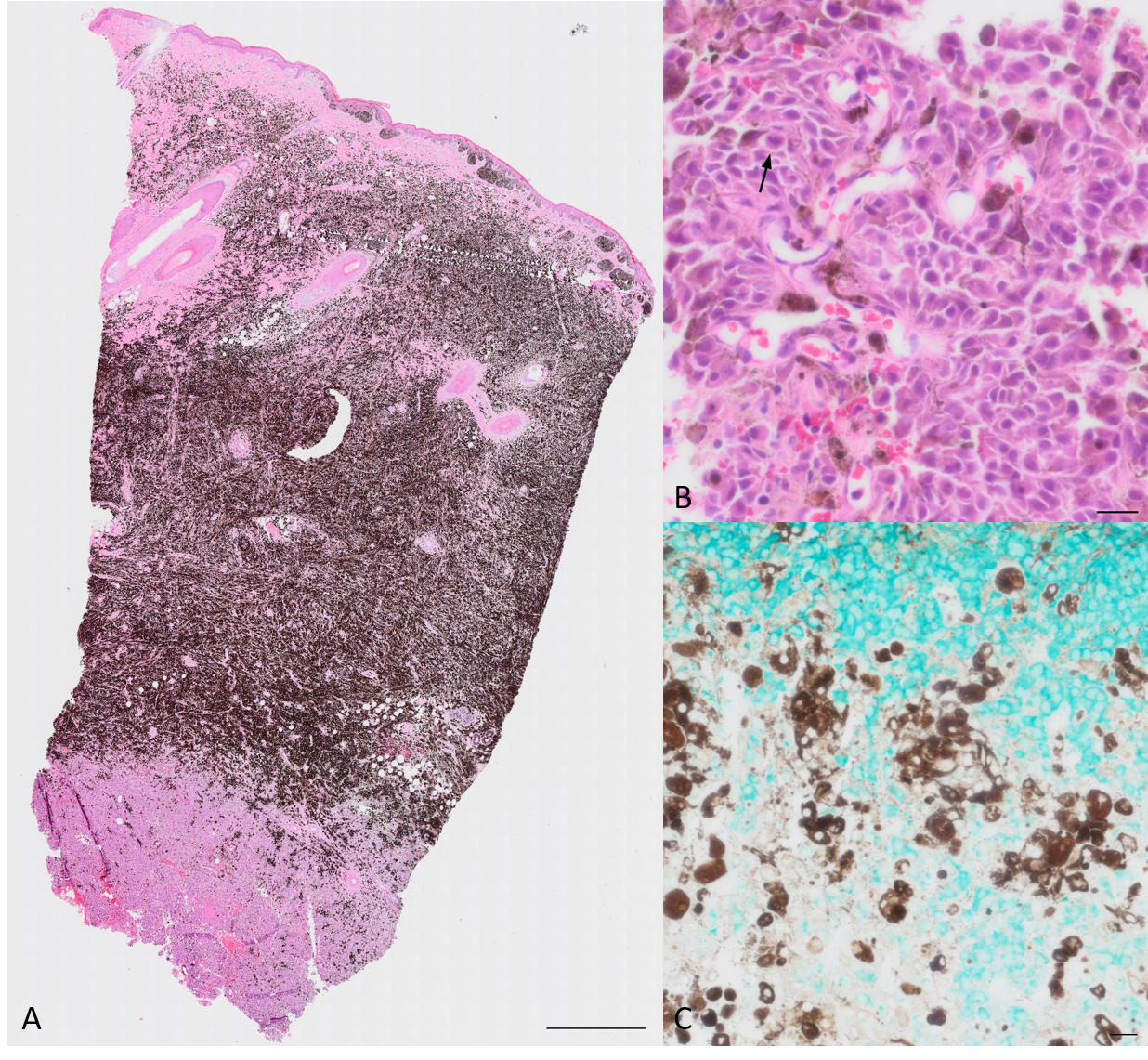


Figure A4a-c: HRP-Pig, biopsy cutaneous melanoma. **A.** Within the superficial dermis and extending into the subcutis parts of a cell-rich, highly pigmented neoplastic mass with a reduced degree of pigmentation towards the subcutis are present. Hematoxylin and eosin stain, bar = 1 mm. **B.** Neoplastic cells are arranged in sheets, medium-sized to large, with either round to polygonal or spindeloid shape, scant to moderate amount of eosinophilic, granular cytoplasm that contains variable amounts of brown-black, granular pigment (melanin) and indistinct cell borders. A mitotic rate of 3 mitoses (arrow) per 2.37 mm^2^ was present. Hematoxylin and eosin stain, bar = 20 µm. **C.** Tumor cells express PNL2 (green-labelled cells) as marker for melanocytes in non-bleached sections. Immunohistochemistry with HistoGreen, scale bar = 20 µm.

References:

1. Becker, K., Kegler, K., von Altrock, A., Kuchelmeister, K., Baumgärtner, W., & Wohlsein, P. (2019). Cutaneous Pigmented Neurofibroma in a Pig - Morphology and Immunohistochemical Profile. Journal of comparative pathology, 168, 25–29. https://doi.org/10.1016/j.jcpa.2019.03.002
